# Home gardens as a predictor of enhanced dietary diversity and food security in rural Myanmar

**DOI:** 10.1186/s12889-019-7440-7

**Published:** 2019-08-20

**Authors:** Anu Rammohan, Bill Pritchard, Michael Dibley

**Affiliations:** 1 Discipline of Economics, University of Western Australia, Perth, 6007 Australia; 2 School of Geosciences, University of Sydney, Sydney, Australia; 3 School of Public Health, University of Sydney, Sydney, Australia

**Keywords:** Home gardens, Myanmar, Food security, Dietary diversity

## Abstract

**Background:**

Home gardens have been found to improve food security and dietary diversity in a wide range of settings. However, there is a need to place home gardens within the larger food and nutrition system landscapes that shape the construction of household diets. Myanmar offers a unique opportunity to study these research questions, given the decades of political isolation, high levels of food insecurity and poor nutrition levels.

**Methods:**

The aim of our paper is to use household survey data from three distinctive agro-ecological settings in rural Myanmar to empirically analyse the role of home gardens in influencing household food insecurity and dietary diversity. Our analysis is based on unique survey data conducted in rural Myanmar. The sample includes 3230 rural households from three States/Districts (Magway, Ayeyarwady and Chin). Using information on two dimensions of food security, a series of variables capturing a household’s self-reported food security status and coping strategies when food is not available; and a measure of household’s dietary diversity based on 24-h recall data, we empirically estimate a household’s probability of being food insecure and the diversity of their diets.

**Results:**

There are statistically significant associations between access to home gardens and measures of food security and improved dietary diversity. In particular, for landless households, the ownership of home gardens/ fruits and vines is statistically significant and is associated with a 6.6 percentage points lower probability of a household having to *change* their diet, and a 7.9 percentage points lower probability of *being in hunger.*

**Conclusions:**

From a policy perspective, our results show that promoting home gardens among vulnerable households can improve food security and dietary diversity among vulnerable rural households in Myanmar.

## Background

Home gardens or kitchen gardens, a common form of food production in many rural communities in developing countries offer great potential for improving household food security and alleviating micronutrient deficiencies. Studies from a wide range of settings have found home gardens to be positively associated with better food security and nutritional diversity [1–6]. Home gardening can directly enhance household food security through providing access to a diversity of nutritionally-rich foods, increased purchasing power from savings on food bills and income from sales of garden products, and fall-back food provision during seasonal lean periods [7]. However, the intricate detail of how home gardens alleviate food security and improve dietary diversity remains empirically and theoretically under-researched, and is likely to differ across settings. It is critical to place home gardens within the larger food and nutrition system landscapes that shape the construction of household diets. This implies investigating the association between socio-economic factors and home gardens, and whether home gardens influence households’ dietary and food security.

Furthermore despite the dominance of agriculture, in many developing countries, rural populations experience poor nutritional outcomes, and lag in measures of social and economic progress. Dietary quality remains poor in many developing countries [8], and food consumption is dominated by cheap, starchy foods and there is limited consumption of energy rich nutrient-dense foods (fruits, vegetables and animal protein) [9]. Increasingly, there is a growing recognition of the need to also account for dietary diversity, by taking into consideration the number of different food groups consumed in a household over a given reference period [10, 11]. Previous research shows that diversity indices reflect overall dietary quality, and is positively associated with measures of food security [12–14]. It is in this context that access to home gardens can play a critical role in improving household food security and dietary diversity.

However, the diversity of livelihood and agro-environmental contexts among communities across different contexts means that the role of home gardens varies across settings.

The aim of this paper is to examine the role of home gardens in alleviating household food insecurity and improving dietary diversity among rural households in Myanmar. Myanmar is a mainly agrarian country with severe problems of rural poverty and malnutrition. To the best of our knowledge the present study is the first detailed quantitative assessment of home gardens in the Myanmar context, given its decades of political isolation. Since 2011 several donor-funded, national surveys have been undertaken, but none specifically asked questions about home gardens. Household respondents were not asked about this topic in the 2011 and 2013 LIFT (“Livelihoods and Food Security Trust Fund”) surveys, which are typically regarded as providing the most extensive and comprehensive databases on the rural conditions of the country.

The data used in this analyses are drawn from a survey of 3320 households from three distinctive agro-ecological settings of rural Myanmar that the authors conducted between February and April 2016. These include the dry zone in the central plains; the fecund delta of the Ayeyarwady River, and the mountainous region of southern Chin State.

Our analysis finds statistically significant associations between access to home gardens and measures of food security and improved dietary diversity, particularly among households without agricultural land holdings.

In the next section, we review previous research on home gardens, followed by Methods section which contains a description of our data and the methods used in the empirical analysis. The main findings from the empirical analyses are presented in Results section, followed by the Conclusions section.

Home gardens can be loosely defined as a “traditional land use system around a homestead, where several species of plants are grown and maintained by the household members and their products are primarily intended for the family consumption” [15].^1^ They exist “in backyards, farmyards, kitchens, containers, small patches of available land, vacant lots, on rooftops and tabletops, and along roadsides and the edges of fields. They are generally close to a house and source of water, and are managed by family members using low-cost inputs” [16]. Given the complexity of home gardens within wider household food production systems, including field-based cropping, the collection of semi-cultivated and wild foods, and the rearing of livestock, it is difficult to precisely articulate the concept beyond general terms [17]. Nevertheless, although the forms and functions of home gardens differ widely across the developing world, they tend to exercise similar social, economic and nutritional roles whatever their settings.

Our conceptual framework for linking home gardens to dietary diversity and food security indicators is developed from recent research into patterns of rural livelihood change in the global South. Smaller proportions of rural populations in the global south today depend on own agriculture as their sole means of sustenance. It is increasingly the norm that households are involved in diverse livelihood activities across subsistence and wage-labour in the agricultural and non-agricultural sectors [18, 19]. This has dramatic impacts on the pathways through which households procure their food, with implications for dietary diversity and quality [20]. As households shift from own-production to market exchange, patterns of nutrition become dependent on what is physically present in local shops and markets. This may widen food choices however also raise consumption of highly-processed, calorie-dense foods hence undermining initiatives to promote healthy diets [21].

These contexts frame contemporary scholarly attention to home gardens. They contribute to household food availability and generate economic and nutritional benefits through direct and indirect pathways. By directly increasing overall food supply, home gardens reduce the need for households to rely on the market to meet their food requirements, thereby freeing up cash for other uses. Furthermore, many of the fruits and vegetables that are typically cultivated in home gardens tend to be both rich in micronutrients and relatively expensive in shops and market, vis-à-vis more economically accessible staples and, increasingly, cheap processed snack foods. The considerable species diversity in home gardens means that although they may not be primary sources of household sustenance, they add important variety to household diets [17]. In Nepal for example, in the wetter, middle hill regions of the country, more than 75% of home gardens had 21 to 50 diverse species per household [15].

A large body of literature has analysed the links between agriculture and agricultural interventions on household nutrition [22]. A 2008 review of 23 studies [23], found home gardens to positively associated with intakes of fruits and vegetables in 14 cases,^2^ improvements in anthropometric measures in six cases, improvements in serum retinol levels (a biomarker of Vitamin A deficiency) in one case, and mixed results or no effects in two cases.^3^ A meta-analysis of 11 interventions promoting home gardens from 1993 to 2000 found evidence of increased intake of fruits and vegetables in eight cases, improvements in anthropometric measures in one case, and indeterminate or negative associations in two cases [24]^.^^4^ A third study reviewing 23 home garden interventions 1995 and 2009 found mixed results, with unclear evidence of the influence of home gardens on diets other health indicators (stunting, wasting, etc) [25].

The overall tendency in the literature is to associate home gardens with superior food intake, diets and nutrition outcomes. With regards to home gardens research, a project initiated by Helen Keller International in rural Bangladesh integrating home gardens, livestock and nutrition education programs [3, 26–28], and a South African initiative to address Vitamin A deficiencies through home gardens [29] have been influential.

Even if home gardens are empirically found to improve food intake, diets or nutrition, questions remain about over how these dynamics play out within different rural communities. In particular, there is a paucity of knowledge on the role of home gardens in addressing food security and improving dietary diversity in rural Myanmar, particularly among vulnerable households.

Around 10% of Myanmar’s population of 60 million is estimated to be below the official food poverty line, with many pockets of high levels of food and nutrition insecurity across various states/regions and villages [30].

## Methods

The data for this study comes from a unique survey of 3230 rural households from three States/Districts each representing distinctive agro-climatic zone of Myanmar. These include: Magway (the Dry Zone, with agriculture dominated by pulses, maize and, in areas adjacent to watercourses, rice), Ayeyarwady (in the fertile Delta region, the traditional rice bowl of the country, and with important fishery resources), and Chin (in the western hilly zone, and has traditionally been regarded as the most food insecure area of Myanmar). In each State/District, two adjacent townships were selected.^5^ These include the townships of Yesagyo and Pakokku (in Magway), Kyaiklet and Maubin townships (in Ayeyarwady), and Mindat and Kanpetlet townships (in Chin). To establish a representative sample for each township, population counts for each township were obtained from the 2014 Myanmar Census, and a Probability Proportional to Size (PPS) method was applied to randomly select 20 villages. In each village, household lists were obtained from local authorities and a random sample of 30 households selected for survey. This method provided a target sample of 600 households per township, or 3600 for the entire survey.

Selected households were interviewed face-to-face by a team of enumerators employed by two local partner institutions, the University of Community Health Magway, and the University of Public Health Yangon, under supervision from the research team. The survey took place between in the lean season February–April 2016, which is in the lean season. Information on home gardens was asked as part of a wider set of questions on household demographics, assets, livelihoods, food security and dietary diversity. It is important to note that all our data is at the household level, as we are unable to account for intra-household differences in allocation of food.

### Empirical strategy

The empirical aim is to analyse the role of access to home gardens on household-level food security and dietary diversity in rural Myanmar. To this end, the first step is to identify measures of household food security and dietary diversity.

#### Measures of food security

We measure food security using a slightly varied application of the Household Food Insecurity Access Scale (HFIAS) methodology, developed by USAID [28]. With a recall period of 4 weeks, this methodology was originally developed for the FANTA (USAID) initiative with the aim of providing a holistic methodology to capture the *experience* of food insecurity [31]. The senior-most knowledgeable female of the household was asked a series of questions on household member’s *experiences* of food insecurity. In particular, questions were asked of household’s food intake and coping strategies in the event of non-availability of food, over the past 4 weeks.

Each respondent’s self-reported assessment of their household’s food security was classified into the following five discrete categories: (i) ‘*Shortage of food*’= 1 if in the last 4 weeks: respondent worried that the household would not have enough food; or any household member had to eat a limited variety of foods; or any household member had to eat a smaller meal than needed; or any household member had to eat fewer meals in a day; or there was ever no food to eat of any kind in the household; or any household member went to sleep hungry at night because of lack of food; or any household member had to go 24 h without eating anything because of lack of food, 0 otherwise. (ii) ‘*Hunger*’ = 1 if at any point in the last 4 weeks, there was no food of any kind in the household; or any household member went to sleep hungry at night because of lack of food; or any household member had to go 24 h without eating anything because of lack of food, 0 otherwise; (iii) ‘*change food*’ = 1 if in the last 4 weeks: any household member had to change their diet to cheaper; or less preferred foods, or were not able to eat the kinds of foods their prefer, because of a lack of resources, 0 otherwise; (iv) ‘*reduce food*’ = 1 if in the last 4 weeks: any household member had to eat a limited variety of foods because of a lack of resources; or eat a smaller meal than they felt was needed, or eat fewer meals in a day, because there wasn’t enough food, 0 otherwise; and (v) ‘*Borrow*’ = 1 if in the past 4 weeks: the household took food on credit from a local shop; or had to borrow food from relatives or neighbours, 0 otherwise.

Responses to (i) and (ii) directly assess levels of household food insecurity, whereas (iii)- (iv) relate to household’s coping strategies in the event of food shortages.

Given that these responses capture different elements of food insecurity, the dependent variable food security is measured separately for each of the five potential food security indicators. Accordingly, we estimate binary choice reduced form univariate Probit models for each of the five food insecurity indicators. Formally, the model can be written in the following general form:

1 $$ Food\ securityi={\propto}_0+{\propto}_1 socio-{econ}_i+\kern0.5em {\propto}_2 land-{ownership\ status}_i+\kern0.5em {\propto}_3 homegarden+{\propto}_4{geographical}_i+{\varnothing}_i $$

Where the dependent variable *Food security*_*i*_ captures the food security in household *i*, the vector socio-econ refers to the socio-economic and demographic characteristics of the household measured using household size and the gender of the household head; household’s economic characteristics are captured using wealth quintiles (based on household assets and calculated using principal components analysis). The household’s land-owning status is measured using a dummy variable that takes on a value of one if the household owned land, 0 otherwise, and for land-owning households, we include categorical variables for land size. We include a dummy variable for whether or not the household has a home garden and the vector geographical includes indicator variables for the six townships in the sample. Further details on the explanatory variables and descriptive statistics are provided below.

#### Measure of dietary diversity

Our next dependent variable is a measure of dietary diversity. As previously discussed, dietary diversity is commonly used as a proxy measure for the quality of human diets [13, 14]. Previous research has found dietary diversity to be positively associated with measures of food security [32, 33]. Using a 24-h food recall methodology [10], self-reported consumption of food items were grouped into ten food groups in accordance with the Minimum Dietary Diversity – Women (MDD-W) methodology [7]. The same household member answering the food security questions also provided information on the food intake of household members. The diversity of household diets is measured as the intake of food from the ten discrete food groups among household members over the previous 24 h, and takes on a value of 1 if anyone in the household consumed those foods in the previous 24 h, 0 otherwise.

The dietary diversity methodology has previously been used to construct the variable *Dietary Diversity Score (DDS)*, which measures the number of unique food groups (rather than number of different foods) consumed by members of the household over the last 24 h [33–35]. We are interested in the diversity of food groups rather than the number of foods because it is possible that a household has consumed a large number of foods, but they may all be from the same food group, thus not providing any diversity in diet.

The ten food groups considered are those with the most density of nutrients, and therefore those most important in diet: starchy staples, beans and peas, dairy, flesh foods, eggs, nuts and seeds, dark, leafy greens (Vitamin A rich), Other fruits and vegetables (Vitamin A rich), Other fruits and other vegetables.

To construct the DDS variable, binary response variables are defined for each of the values taken by the DDS variable. A household is classified as being in the category DDS2 if household members consumed at least one food from two different food groups, and zero otherwise; a household is defined as having a DDS3 if it consumed at least three of the food items and zero otherwise and so on. In our sample, we observe that on average households in Chin ate from 3.5 food groups; households in Ayeyarwady from 3.9 food groups and households from Magway from 4.9 food groups. The DDS is clustered between 2 and 5 food groups, with DDS2 being the worst outcome, and DDS5 being the best outcome. Since nearly all the households in our sample consumed from at least two food groups, and given the natural ordering of the DDS variable, we use the Ordered Probit model for our empirical analyses.

Specifically, following previous research [34], we categorise the DDS into four categories: DDS2, DDS3, DDS4 and DDS5, where DDS2 is the lowest category of dietary diversity. The food consumption categories are represented by an ordered variable V that assumes the discrete ordered values of 0, 1,.. .and j. The ordered probit model for V (conditional on explanatory variables x) can be derived from a latent variable model.

Assume that the latent variable D* is determined by D* = x0b + e, where x is a vector of household’s socioeconomic and community-level characteristics entering the equation and e refers to the error term, which we assume is normally distributed across observations.

However, D*, the propensity to consume from a particular food group, is unobserved. Given that we observe D, the household’s dietary diversity status, the observed aspects of a household’s dietary diversity status can formally be written as:

2 $$ D=\left\{\begin{array}{c}0\  if\ only\ 2\  food\ groups\  are\  consumed\\ {}1\  if\ 3\  food\ groups\  are\  consumed\\ {}2\  if\ 4\  food\ groups\  are\  consumed\\ {}3\  if\ 5\  or\ more\ food\ groups\  are\  consumed\end{array}\right. $$

and each of these categories is a discrete category of the dependent variable, which can be explained by the same set of explanatory variables.

### Explanatory variables

The key explanatory variable used in this paper is a measure of whether the household had access to a home garden.

### Home gardens

Questions about home gardens were incorporated into the questionnaire as part of a larger group of questions about household food production. We sought to elicit as full a description as possible about households’ food production activities by asking respondents if they: (i) had land (owned or leased) upon which they grew crops or grazed livestock; (ii) had fruit trees or vines; (iii) owned livestock for food purposes in or around their homestead; (iv) had a ‘home garden’ (defined as growing crops, fruits, or vegetables) in or around their homestead; (v) caught or collected wild animals, fruits or other foods from forests, common land or rivers, lakes, etc. This wider context is relevant, because in some agro-ecological settings, distinctions between home gardens and households’ other food production activities can be blurred [36].

Specifically, respondents were asked if they owned a home garden, their access to fruit trees and vines, and their ownership of livestock. Using these responses, we combine home gardens & fruits and vines into one variable, which is categorised as follows: = 1 if yes, and 0 if no. This is because they both represent responses on non-agricultural production. However, we create a separate categorical variable for livestock ownership. This is because during the qualitative survey which followed the household data survey we realised that there was some confusion in respondents’ answers to these questions. For example, fruit trees were sometimes counted in home gardens, so in the empirical analyses we combined the trees and vines variable with the home gardens variable.

Although livestock ownership may be complementary to home gardens if animals provide a source of manure and natural weeding, domestic animals eat and trample produce, in which case households may face a trade-off between keeping livestock and maintaining home gardens.^6^

Furthermore, for those households that had access to home gardens/ fruits and vines, we asked respondents to nominate the crops grown in their home gardens/ fruits & vines. We used these responses to construct indicator measures of home garden crop diversity- ranging from home garden crop diversity 0 (no diversity in home garden crops grown) to home garden diversity 3 (indicating that the household grew three or more diverse crops in their home gardens).

From Table 4 we note that on average our households grow over two home garden crops, except in Maubin township where they grow just under two home garden crops.

We also include a set of variables to capture the household’s demographic and socio-economic characteristics. These include household socio-economic and demographic characteristics including variables such as the household head’s sex and household size; economic status variables measured where using data on asset ownership for each household we create a wealth index using principle components analysis. The households in the sample are then categorized into one of five wealth quintiles (ranging from poorest to richest households). These wealth quintiles provide a more permanent measure of household wealth that is less affected by transitory income changes, and is less likely to be subject to measurement error compared to income.

Additionally, we include information on household land ownership, which is defined as aggregate total land operated by all household members. In these questions, ‘land ownership’ is defined as being under the direct control of any household member, without the obligation to pay rent or a share of production. In this sense, households may either have formal title to the land through government documentation, or land could be held without formal title but via de facto possession. Our interest is not in the legality of tenure, but in the economic principle of a household having access to land. Therefore, for landholding households (i.e., those answering ‘yes’ to the question of whether they own land), we ask the respondent to indicate the total area of land that is owned by all members of the household. We include four indicator variables, no land, land area < 2 acres, land area between 2 and 5 acres, and land area ≥ 5.

Note that all land in Myanmar is owned by the state and cultivators only retain the tilling rights, and land-use rights can now be sold, mortgaged or inherited. This pattern of intergenerational land transfer has increased land fragmentation of holdings and small farming households. Although the new land laws of “Vacant, Fallow and Virgin Land Management Law” and “Farmland Law” were both enacted in March 2012 to solve land related problems, land reform continues to be a major challenge [37].

Finally, we include variables to capture geographical differences across our study sites, by including indicator variables for the six study townships: Yesagyo and Pakokku (in Magway), (Kyaiklet and Maubin townships (in Ayeyarwady), and Mindat and Kanpetlet townships (in Chin).

## Results

### Descriptive statistics

The descriptive statistics for the variables included in the empirical analysis are presented in Tables 1 and 2. Table 1 presents the descriptive statistics classified by whether or not the household had access to home gardens; and in Table 2 we present descriptive statistics disaggregated by DDS categories. The main point to note from Table 1 is that 959 households have access to home gardens out of our sample of 3239 households. Furthermore, 38% of households with home gardens do not own agricultural land, and 16% of households with home gardens are in the poorest wealth quintile.

**Table 1:** Descriptive statistics- comparison of households with home gardens and those without

Variable	HHs with home-garden & fruits & vines	HHs without home-garden & fruits & vines	t-statistic
	Mean	Mean	
Dietary Diversity Score	4.09	4.08	0.00
Food Security			
Change	0.63	0.64	−0.02
Reduce	0.64	0.61	0.03
Shortage	0.75	0.72	0.03
Hunger	0.10	0.13	−0.03*
Borrow	0.67	0.70	−0.03
Explanatory variables			
Land Area: < 2 acres	0.17	0.11	0.06***
Land Area: 2–5 acres	0.29	0.17	0.11***
Land Area: > 5 acres	0.16	0.11	0.05***
Household owns no land	0.38	0.60	−0.22***
Household owns livestock	0.65	0.48	0.17***
Home garden diversity score: 0	0.28	1.00	−0.72***
Home garden diversity score: 1	0.23	0.00	0.23***
Home garden diversity score: 2	0.19	0.00	0.19***
Home garden diversity score: 3	0.14	0.00	0.14***
Home garden diversity score: 4	0.14	0.00	0.14***
Wealth quintile: Poorest	0.16	0.22	−0.05***
Wealth quintile: Poor	0.19	0.21	−0.01
Wealth quintile: Middle	0.19	0.20	−0.01
Wealth quintile: Rich	0.21	0.19	0.02
Wealth quintile: Richest	0.24	0.18	0.06***
Mindat	0.18	0.14	0.04**
Kanpetlet	0.20	0.11	0.09***
Pakokku	0.08	0.23	−0.14***
Yesagyo	0.15	0.17	−0.02
Kyaiklet	0.22	0.17	0.05**
Maubin	0.16	0.18	−0.02
Observations	959	2280	3239

the numbers refer to mean proportions

*, ** and *** refer to statistical significance at 10%, 5% and 1% levels respectively

**Table 2:** Summary statistics by food security indicators

Variable	DDS 2	DDS 3	DDS 4	DDS 5
Food security				
Change	0.78	0.72	0.65	0.50
Reduce	0.74	0.71	0.64	0.48
Shortage	0.87	0.79	0.75	0.61
Hunger	0.23	0.16	0.11	0.05
Borrow	0.80	0.75	0.71	0.58
*Explanatory variables*				
Township: Mindat	0.21	0.21	0.16	0.09
Township: Kanpetlet	0.20	0.15	0.15	0.08
Township: Pakokku	0.07	0.16	0.19	0.25
Township: Yesagyo	0.04	0.11	0.15	0.28
Township: Kyaiklet	0.23	0.19	0.19	0.16
Township: Maubin	0.24	0.19	0.16	0.15
Land owned: Less than 2 acres.	0.56	0.52	0.53	0.53
Land owned: 2–5 acres	0.13	0.15	0.12	0.13
Land owned: 5 or more acres.	0.21	0.24	0.21	0.18
Household owns no land	0.56	0.52	0.53	0.53
Household has a home garden/fruits and vines.	0.17	0.22	0.24	0.22
Household owns livestock.	0.61	0.56	0.57	0.45
Home garden diversity score: 0 (no diversity)	0.83	0.79	0.76	0.78
Home garden diversity score: 1 (2 diverse crops)	0.07	0.08	0.08	0.06
Home garden diversity score: 2 (3 diverse crops)	0.05	0.06	0.06	0.06
Home garden diversity score: 3 (4 or more diverse crops)	0.05	0.08	0.10	0.10
Wealth quintile: Poorest	0.38	0.22	0.19	0.11
Wealth quintile: Poor	0.25	0.25	0.21	0.14
Wealth quintile: Middle	0.19	0.23	0.20	0.19
Wealth quintile: Rich	0.11	0.19	0.23	0.24
Wealth quintile: Richest	0.08	0.12	0.18	0.33

the numbers represent mean proportions

In general food insecurity is high in our sample, and there is a strong association between food insecurity measures and DDS. In particular, over three-quarters of the households that report having to change their diet, reduce their food intake, or face food shortage are also categorized as being in the lowest DDS category (DDS2). Furthermore, 87% of the households in DDS2 category report a shortage of food. Indeed this is 61% even among households in DDS5 category 5, where over half the sample reporting having to change their diet due to a lack of resources, suggesting a high degree of seasonality. Surprisingly on average, only 23% of the households report being in hunger in DDS2 category (5% among the households in the highest dietary diversity category- DDS5). However, it is likely that households are taking measures to address their hunger when they face food shortages. For example, a high proportion of the households report having to change their diets, borrow or reduce their food intake due to lack of resources.

Not surprisingly, dietary diversity is highest among the wealthiest households, with approximately 51% of the households in the wealthiest quintile being in the two highest DDS categories.

There are also regional differences across our sample, with households in the townships of Pakokku and Yesagyo (Magway state) having the most diverse diets (DDS5). Together these two townships account for over half the households represented in the DDS5 category. On the other hand, the townships of Maubin and Kyaiklet (Ayeyarwaddy) have the highest proportion of households in the lowest DDS category, and together they represent approximately 48% of the households in DDS2 category. Households in Mindat, Kanpetlet, Maubin and Kyaiklet have at least 20% of the households surveyed reporting DDS = 2.

The unconditional means also show that access to home gardens is associated with better dietary diversity, with 22% of the households in the highest DDS category (DDS5) owning a home garden compared to 17% in the lowest DDS category (DDS2). In terms of the links between land ownership and home gardening, we note that only 12% of landless households have access to a home garden, compared to 31% among landed households.

In Table 3 we present land ownership patterns by township. It is unsurprising that land sizes are the largest in the two townships in the fertile Ayeyarwaddy delta, where on average a household in Kyaiklet township owns approximately 7.34 acres of land. However, it is noteworthy that while the average land size is large in these townships, nearly 63% of households in our sample do not own land in the township of Kyaiklet, and 75% do not own land in Maubin in the Ayeyarwaddy delta. Together with Table 2 they indicate high levels of food insecurity in these Ayeyarwaddy townships which also have high levels of landless households, and large inequities in land ownership.

**Table 3:** Land holding patterns by Township

Variable	Chin		Magway		Ayeyarwaddy	
	Mindat	Kanpetlet	Pakokku	Yesagyo	Kyaiklet	Maubin
Total land owned by household (Mean)	3.12	2.80	5.53	3.87	7.34	5.48
Household owns no land.	24.09	25.45	65.77	55.19	62.98	75.13
Less than 2 acres.	27.33	19.09	11.13	15.56	4.02	5.91
2–5 acres	42.51	46.59	8.43	16.11	12.23	8.35
5 or more acres.	6.07	8.86	14.67	13.15	20.77	10.61
Observations	494	440	593	540	597	575

On the other hand, in the townships of Mindat and Kanpetlet in the mountainous Chin state, land sizes are small with households owning on average just 3.12 and 2.80 acres of land, respectively. Moreover, the proportion of households reporting no land ownership is relatively low (approximately 25%). It is important to point out that land ownership patterns in these two townships are a bit complicated as they are not a major agricultural growing region in Myanmar, and land is sometimes communally owned, and it is unclear whether we are observing land ownership or cultivation rights.

The situation in the townships of Yesago and Pakokku (in Magway) are similar to the Ayeyarwaddy townships, albeit with lower levels of landlessness.

To get a better sense of home garden ownership, in Table 4 we report ownership patterns for home gardens, fruits and vines, and livestock across our townships. We observe that on average there are just over 2 home garden crops grown in township, but the average number of crops grown differ across townships, with 2.6 different crops grown in Yesagyo (in Magway) and just 1.9 crops in Maubin (in the Ayeyarwady). Ownership of home gardens ranges between a high of 34.8% in Kanpetlet township (Chin state) to a low of 10% in Pakokku. However, home gardens are often complementary to livestock ownership and are substitutes with fruits and vines, as together they account for informal food growing patterns. We observe that across our sample, approximately 83% of the households in Mindat and Kanpetlet (in Chin state) have access to home gardens, fruits and vines and livestock, and below 50% in the two Magway townships (34.2% in Pakokku and 48.5% in Yesagyo). The townships in Ayeyarwaddy exhibit similar patterns to the Magway townships with regards to home garden access. Our results show that 21 and 15% of the households in Kyaiklet and Maubin, respectively have access to just home gardens, and approximately 74 and 66% have access to either of home gardens/ fruits and vines or livestock.

**Table 4:** Ownership of Kitchen Garden, Fruits and Vines, and Livestock by Township

Variable	Mindat	Kanpetlet	Pakokku	Yesagyo	Kyaiklet	Maubin
Only Own Home garden^a^	0.26	0.35	0.10	0.25	0.21	0.15
Own Home garden and Fruits & Vines	0.35	0.44	0.13	0.27	0.35	0.28
Own Home garden, Fruits & Vines and Livestock	0.87	0.83	0.34	0.49	0.74	0.66
Average number of crops per home garden	2.5	2.4	2.2	2.6	2.1	1.9
Observations	494	440	593	540	597	575

^a^The figures refer to mean proportions

However, these unconditional means do not provide a full picture of the links between food security, dietary diversity and access to home gardens. In the next section, we present the main estimation results of our analyses (Tables 5, 6 and 7).

**Table 5:** Univariate Probit Estimations results for landless households- Food Security

Variables	(1)	(2)	(3)	(4)	(5)
	Change Marg. Eff	Reduce Marg. Eff.	Shortage Marg. Eff.	Hunger Marg. Eff.	Borrow Marg. Eff.
Mindat	−0.127**	− 0.230***	− 0.152***	− 0.014	− 0.079
	[0.057]	[0.061]	[0.055]	[0.056]	[0.068]
Kanpetlet	−0.108**	− 0.108**	− 0.057	− 0.190***	0.220***
	[0.043]	[0.043]	[0.037]	[0.043]	[0.053]
Pakokku	−0.191***	− 0.226***	− 0.180***	− 0.172***	0.126**
	[0.046]	[0.047]	[0.041]	[0.045]	[0.056]
Yesagyo	−0.360***	− 0.354***	− 0.235***	− 0.173***	0.138***
	[0.045]	[0.045]	[0.041]	[0.042]	[0.053]
Kyaiklet	−0.280***	−0.300***	− 0.156***	− 0.146***	0.152***
	[0.043]	[0.044]	[0.038]	[0.042]	[0.052]
Household Size	0.021***	0.017**	0.019***	0.010***	0.024***
	[0.007]	[0.007]	[0.006]	[0.004]	[0.006]
Head of Household: Male	−0.070	− 0.020	0.061	0.069	0.044
	[0.080]	[0.081]	[0.069]	[0.057]	[0.072]
Own Livestock	0.014	0.060**	0.023	−0.007	0.019
	[0.026]	[0.026]	[0.023]	[0.015]	[0.024]
Own Home gardens/ Fruits & Vines	−0.066**	0.022	0.034	−0.079***	−0.003
	[0.030]	[0.030]	[0.027]	[0.019]	[0.027]
Wealth Quintile: Poor	−0.081***	−0.130***	− 0.064***	−0.069***	− 0.056**
	[0.031]	[0.030]	[0.023]	[0.026]	[0.025]
Wealth Quintile: Middle	−0.190***	− 0.223***	− 0.197***	− 0.092***	− 0.117***
	[0.035]	[0.034]	[0.030]	[0.026]	[0.029]
Wealth Quintile: Rich	−0.239***	− 0.314***	− 0.273***	− 0.141***	− 0.231***
	[0.038]	[0.037]	[0.033]	[0.025]	[0.034]
Wealth Quintile: Richest	−0.382***	− 0.482***	− 0.444***	−0.171***	− 0.442***
	[0.039]	[0.038]	[0.037]	[0.023]	[0.037]
Observations	1727	1727	1727	1727	1727

Standard errors in brackets; *** *p* < 0.01, ** *p* < 0.05, * *p* < 0.1. Marginal effects are reported

**Table 6:** Ordered Probit Estimation results for landless households- Dietary Diversity

VARIABLES	(1)	(2)	(3)	(4)
	DDS2	DDS3	DDS4	DDS5
Kanpetlet	−0.070**	− 0.029*	0.020*	0.092**
	[0.035]	[0.015]	[0.011]	[0.046]
Pakokku	−0.124***	− 0.069***	0.023**	0.191***
	[0.029]	[0.013]	[0.010]	[0.038]
Yesagyo	−0.155***	− 0.102***	0.015	0.265***
	[0.029]	[0.015]	[0.011]	[0.041]
Kyaiklat	−0.051*	−0.019**	0.016	0.064*
	[0.029]	[0.010]	[0.011]	[0.035]
Maubin	−0.041	−0.014	0.014	0.049
	[0.029]	[0.009]	[0.011]	[0.034]
Household Size	0.001	0.001	−0.000	−0.002
	[0.003]	[0.002]	[0.000]	[0.006]
Head of Household: Gender	0.065*	0.044*	−0.008	−0.110*
	[0.039]	[0.026]	[0.005]	[0.065]
Own Livestock	0.009	0.006	−0.001	− 0.015
	[0.012]	[0.008]	[0.001]	[0.021]
Own Home garden and Fruits & Vines	−0.077**	− 0.052**	0.009*	0.131**
	[0.039]	[0.026]	[0.005]	[0.065]
Home garden diversity score: 0	−0.005	−0.004	0.001	0.009
	[0.029]	[0.020]	[0.003]	[0.050]
Home garden diversity score: 1	−0.014	− 0.010	0.001	0.024
	[0.030]	[0.022]	[0.002]	[0.053]
Home garden diversity score: 2	−0.038	− 0.029	0.001	0.070
	[0.036]	[0.032]	[0.003]	[0.074]
Home garden diversity score: 3	−0.079***	− 0.074*	− 0.011	0.173**
	[0.030]	[0.038]	[0.016]	[0.086]
Wealth Quintile: Poor	−0.044**	−0.018**	0.013**	0.057**
	[0.019]	[0.008]	[0.006]	[0.025]
Wealth Quintile: Middle	−0.070***	−0.033***	0.018***	0.098***
	[0.020]	[0.010]	[0.005]	[0.028]
Wealth Quintile: Rich	−0.101***	−0.057***	0.019***	0.154***
	[0.020]	[0.012]	[0.005]	[0.031]
Wealth Quintile: Richest	−0.161***	−0.124***	− 0.002	0.309***
	[0.018]	[0.015]	[0.009]	[0.035]
Observations	1727	1727	1727	1727

Standard errors in parentheses; *** *p* < 0.01, ** *p* < 0.05, * *p* < 0.1. Marginal effects are reported

**Table 7:** Estimation results – full sample

Variables	PANEL A: PROBIT MODEL Dependent variables: Measures of food security				
	Change	Reduce	Shortage	Hunger	Borrow
Land size < 2 acres	0.020	0.012	0.021	−0.009	0.003
	[0.029]	[0.029]	[0.025]	[0.016]	[0.026]
Land size: 2–5 acres	−0.018	− 0.016	−0.018	− 0.031**	−0.020
	[0.027]	[0.027]	[0.024]	[0.013]	[0.024]
Land size: ≥ 5 acres	−0.077**	−0.091***	− 0.074***	−0.067***	− 0.078***
	[0.030]	[0.031]	[0.027]	[0.013]	[0.028]
Own Livestock	0.055***	0.076***	0.046***	−0.011	0.054***
	[0.019]	[0.020]	[0.017]	[0.010]	[0.018]
Own Home garden/Fruits & Vines	−0.032	0.032	0.035**	−0.030***	0.000
	[0.021]	[0.020]	[0.018]	[0.010]	[0.019]
Land size < 2 acres* Home garden	−0.072***	−0.119***	− 0.059***	−0.060***	− 0.068***
	[0.024]	[0.024]	[0.019]	[0.019]	[0.022]
Land size 2–5 acres * Home garden	−0.159***	−0.197***	− 0.155***	−0.089***	− 0.105***
	[0.026]	[0.026]	[0.022]	[0.019]	[0.023]
Land size ≥5 acres * Home garden	−0.196***	−0.265***	− 0.225***	−0.130***	− 0.220***
	[0.027]	[0.027]	[0.023]	[0.018]	[0.025]
	PANEL B: ORDERED PROBIT MODEL Dependent variable: Dietary diversity scores				
	DDS2	DDS3	DDS4	DDS5	
Land size < 2 acres	−0.006	0.028	−0.025	−0.004	
	[0.021]	[0.025]	[0.024]	[0.029]	
Land size: 2–5 acres	−0.017	0.027	−0.022	0.010	
	[0.018]	[0.023]	[0.022]	[0.026]	
Land size: ≥ 5 acres	0.009	−0.034	0.032	−0.013	
	[0.023]	[0.025]	[0.026]	[0.028]	
Own Livestock	−0.000	−0.005	0.037**	−0.035*	
	[0.014]	[0.017]	[0.017]	[0.019]	
Home garden/ Fruits &Vines	−0.049***	0.005	0.047***	0.006	
	[0.013]	[0.018]	[0.018]	[0.020]	
Land size < 2 acres* Home garden	−0.081***	0.035	0.023	0.048*	
	[0.022]	[0.024]	[0.024]	[0.025]	
Land size 2–5 acres * Home garden	−0.104***	0.022	0.011	0.106***	
	[0.023]	[0.025]	[0.024]	[0.026]	
Land size ≥5 acres * Home garden	−0.158***	−0.011	0.046*	0.160***	
	[0.022]	[0.026]	[0.026]	[0.027]	
Observations	3239	3239	3239	3239	3239

Marginal effects are reported. Regressions include all the variables included in previous regressions

Standard errors in parentheses; *** *p* < 0.01, ** *p* < 0.05, * *p* < 0.1

### Role of home gardens on food security and dietary diversity of non-agricultural land-owning households

We are interested in explaining if access to home gardens improves food security and dietary diversity among vulnerable households. This is likely to be particularly critical for the sample of non-land owning households, who are likely to be among the most vulnerable. Therefore, we begin by presenting the estimation results for the Probit and Ordered Probit models for the sample of non-agricultural land owning households, in Tables 5 and 6 respectively. The Univariate Probit estimations are presented separately for each of our five food security indicators, for the sample of non-agricultural land owning households. For ease of interpretation we present marginal effects, which show the percentage change in the probability of the outcome variable when the value of a regressor changes, holding all other variables constant.

From Table 5 we observe that for landless households, the ownership of home gardens/ fruits and vines is statistically significant and is associated with a 6.6 percentage point lower probability of a household having to *change* their diet, and a 7.9 percentage point lower probability of *being in hunger*, with no statistically significant influence on the other food security indicators.

Household wealth is found to play a statistically significant and positive role in mitigating food insecurity. Relative to households in the poorest wealth quintile, households from each of the higher wealth quintiles have a lower probability of being food insecure, across each of our food security indicators, with the size of the association increasing monotonically with each higher wealth quintile, with the largest effects observed for the wealthiest households. In particular, relative to a household in the lowest wealth quintile, a household in the highest wealth quintile has a 38.2 percentage points lower probability of reporting ‘change’, a 17.7 percentage points lower probability of reporting ‘hunger’, and a 44.4 percentage point lower probability of reporting a shortage of food.

There are also regional differences across our sample. Relative to Maubin township, where 75% of the households had no land, we observe that households living in the other townships had a statistically significant and negative association with reporting needing to ‘change their diet’, having to reduce their food consumption, face ‘shortages’ or ‘hunger’.

In Table 6, we present the Ordered Probit estimation results for dietary diversity, using the DDS categories as our dependent variable and the same set of explanatory variables as with the food security regressions. Since our data has information on the diversity of fruits and vegetables grown in home gardens, we additionally include among our explanatory variables indicator variables for the diversity of home garden crops (according to FAO classification). These variables capture information on whether the household’s home garden includes cultivation across one food group, two, three or more.

From Table 6 we observe that in the sample of non-agricultural land owning households, access to home gardens/ fruits and vines is strongly associated with better DDS. More specifically, access to home garden garden/ fruits & vines is associated with a 7.7 percentage points lower probability of being in the lowest DDS category (DDS 2) and a 13.1 percentage points higher probability of being in the highest DDS category, relative to a household with no home gardens.

Not surprisingly, having greater diversity in home garden crops grown also improves dietary diversity, with growing three or more diverse crops in the home garden being associated with a 7.9 percentage points lower probability of being in DDS category 2 and a 17.3 greater probability of being in the highest dietary diverse category (DDS 4). There is no statistically significant correlation between home garden crop diversity and dietary diversity for three or less groups.

## Discussion

We observe that after controlling for all other characteristics, there is a statistically significant and monotonically increasing relationship between greater dietary diversity and household wealth. In particular, relative to a household in the lowest quintile, a household in the second quintile has a 5.7 percentage points higher probability of being in DDS4 and a 4.4 percentage points lower probability of being in the lowest DDS category. This positive association between household wealth and dietary diversity is particularly large for households in the highest wealth quintile, who have a 30.9 percentage points higher probability of being in DDS5 relative to a household in the lowest wealth quintile.

### Does access to home gardens improve food security and dietary diversity for agricultural land-owning households?

The above section found a positive association between home gardens and measures of food security and dietary diversity. To understand if home gardens play a similarly important role in the full sample (including both land owning and non-land owning households), we additionally control for the influence of land size using four discrete land ownership categories- no agricultural land, land below 2 acres, land between 2 and 5 acres, land over 5 acres. These results are presented in Table 7 for Probit and Ordered Probit models reporting marginal effects for food security indicators and dietary diversity score (DDS), in Panel A and B respectively.

From Panel A, it is noteworthy that despite controlling for household wealth, relative to households with no land, for the largest land-owners is significantly associated with a lower probability of reporting food insecurity across all our five measures. Specifically, relative to a household with no land, ownership of over 5 acres of agricultural land reduces the probability of a household reporting the need to ‘change’ their diet by 7.7 percentage points, with a 6.7 percentage points lower probability of ‘hunger’, a 7.4 percentage points lower probability of experiencing food shortages and a 7.8 percentage points lower probability of needing to reduce food intake. These results are in keeping with previous research from Myanmar which used the LIFT dataset, and found that agricultural land ownership plays a key role in reducing food insecurity [30]. However, their study did not take into account the role of home gardens.

However, home garden access is only statistically significant and negatively associated with the ‘hunger’ indicator for food insecurity, where we observe that a household with home gardens has a 3 percentage points lower probability of reporting hunger. Surprisingly, we find a positive and statistically significant association between access to home gardens and food shortage. Since our dataset is a cross-section, it is difficult to infer causality, and it is possible that households participate in home garden cultivation when they face food shortages.

Notably, from Panel B we observe that land size has no statistically significant influence on dietary diversity. However, home gardens are positively and significantly associated with greater dietary diversity. In particular, we observe that a household with access to home gardens has a 4.9 percentage points lower probability of being in the lowest dietary diversity category (DDS2), and there is a 4.7 percentage point positive association between home garden access and being in the second highest dietary diverse category.

### Robustness tests

In the previous sections, we demonstrated the positive associations between measures of food security and dietary diversity and access to home gardens. Since home gardens may have a differential influence on households depending on the size of their land holdings, we include some additional interaction terms in our regressions. In particular, we interacted the variable home gardens access with different categories of land size, both for the food security and dietary diversity regressions. These interaction terms are statistically significant and have the expected signs, both in models for food security and dietary diversity. In other words, home gardens reduce food insecurity and improve dietary diversity, and the higher the size of agricultural land, the greater the role that home gardens play in reducing food insecurity and improving dietary diversity.

The results for the other variables are as expected, with higher wealth quintiles being associated with better dietary diversity and food security.

## Conclusions

There is wide acknowledgement of the critical role played by home gardens in addressing the food security and dietary needs to rural populations in developing country settings. The aim of this paper was to analyse the role of access to home gardens on household-level food security and dietary diversity in rural Myanmar. Myanmar offers a unique opportunity to study these research questions, given the decades of political isolation, high levels of food insecurity and poor nutrition levels. To the best of our knowledge the present study is the first detailed assessment of home gardens in the Myanmar context.

Using information on two dimensions of food security, a series of variables capturing a household’s self-reported food security status and coping strategies when food is not available; and a measure of household dietary diversity based on 24-h recall data, we empirically estimate a household’s probability of being food insecure and the diversity of their diets. Our analysis has three key findings. Firstly, in the sample of landless households, access to home gardens is statistically significant and negatively associated with food insecurity (measured using change and hunger); and positively associated with higher dietary diversity. Secondly, an improvement in the diversity of crops grown in the home garden improves dietary diversity. Finally, access to home gardens is also significantly associated with lower food insecurity and better dietary diversity, however, the magnitude of the effects is larger for the largest landowners.

Recent research has pointed to home gardens as a playing vital role for households in the context of far-reaching food system transformations [21]. They help maintain consumption of diverse, healthy foods in situations where emergent pathways of food availability favour unhealthy contributors to diets and in contexts where poor households have limited access to agricultural land, non-farm livelihoods to support a diverse diet. Our results show that promoting home gardens among vulnerable households can improve food security and dietary diversity among rural households in traditional food systems such as Myanmar.

## Data Availability

The datasets used and/or analysed during the current study are available from the corresponding author on reasonable request.

## Abbreviations

DDS: Dietary diversity score
FANTA: Food and Nutrition Technical Assistance
HFIAS: Household Food Insecurity Access Scale
LIFT: Livelihoods and Food Security Trust Fund
MDD-W: Minimum Dietary Diversity – Women

## Footnotes

1 Home gardens can also go by the names homestead garden, backyard garden, or kitchen garden. We treat these as synonymous. It also needs noting that our usage of the term ‘home gardens’ is different to that used in some African contexts where it denotes small-scale agroforestry and livestock practices at the village level.

2 Some of these studies focused only on Vitamin A-rich fruits and vegetables.

3 3 There are 29 studies in their meta-analysis, but four assessed the effects of livestock interventions only, and three assessed nutrition education only. Of the remaining 23 studies, all involved a home garden intervention, either singly or in combination with nutrition education or livestock.

4 This study includes reference to a total of 27 intervention studies, of which 11 specifically refer to home gardens.

5 ‘Townships’ are the third tier of government administration in Myanmar, and outside of the larger cities they typically comprise a region characterized by a rural population dispersed in villages and hamlets and an adjacent service town.

6 Note that our survey question on home gardens did not explicitly ask whether output was for own-consumption or sale. However, a prior question in the survey asked respondents to list any crop, fruit or vegetable they produced for sale, so we can infer that all the data we collected on home garden foods were for own-consumption.
